# Molecular Pathogenesis of Cardiac Arrhythmia

**DOI:** 10.3390/biom12101393

**Published:** 2022-09-29

**Authors:** Yosuke Okamoto, Kyoichi Ono

**Affiliations:** Department of Cell Physiology, Akita Graduate School of Medicine, Hondo 010-8543, Japan

The heart is a necessary organ for sustaining life in mammals, and it is the first organ to function during early development. Once this primary engine begins to operate, it continues to pump blood throughout the body until the individual animal dies. The miraculous homeostasis of the heartbeat, which involves tremendous energy consumption, is naturally synchronized with systemic homeostasis. The minimum unit of energy in the body is called the ATP, which is produced in large quantities in organelles called mitochondria. Recently, it has been shown that the quality maintenance of cardiac mitochondria is achieved by mobilizing the immune system [1]. In other words, cardiac mitochondrial function is being addressed as a systemic challenge. In this Special Issue, Dr. Takeuchi and Prof. Matsuoka provide a review focusing on Ca^2+^ dynamics in cardiac mitochondria [2]. Mitochondrial biophysical variables inside the cell are indirectly measured by selective drugs, selective fluorescent reagents, and selective genetic modification. Sophisticated studies have shown that mitochondrial ATP production and mitochondrial Ca^2+^ concentration are closely linked. During heart contraction, the mitochondrial Ca^2+^ concentration and NADH oxidation rate increase synchronously. Without a mechanism to excrete increased intramitochondrial Ca^2+^, the Ca^2+^ overload causes excessive oxidative phosphorylation, which produces reactive oxygen species (ROS); this, in turn, induces ventricular arrhythmias. The Na^+^/Ca^2+^/Li^+^ exchanger (NCLX) is responsible for mitochondrial Ca^2+^ excretion. While NCLX is thought to contribute to mitochondrial homeostasis, chronic activation of NCLX in heart failure conditions, where ATP production has shifted from being fatty acid dependent to being carbohydrate dependent, is expected to result in mitochondrial Ca^2+^ deprivation. Thus, the evaluation of disease status using mitochondrial function is critical and, at the same time, largely complicated. In order to understand such complex biological phenomena, Dr. Takeuchi and Prof. Matsuoka emphasize the importance of mathematical model analysis using computers. 

Dr. Tsumoto and Prof. Kurata reviewed the bifurcation analysis on the cardiac action potentials, a type of mathematical model analysis [3]. In the bifurcation analysis, a set of differential equations (dynamic equations) is considered a system, and a sudden change from one stable status to another stable status is referred to as a bifurcation. The highly abstract approach of describing the action potential as a system enables us to identify specific single molecules that are essential for the stability of the system. Dr. Tsumoto and Prof. Kurata present various bifurcation analyses of cardiomyocyte excitation phenomena. While their review is effectively illustrative of the power of bifurcation analysis, they also identify a problem with bifurcation analysis, namely, that the interpretation of the bifurcations is not simple. For example, in analyzing early after-depolarization (EAD) by reducing the conductance of I_Ks_ and I_Kr_, more than four bifurcations have been observed. Each bifurcation is an arrhythmia with a different molecular mechanism. In addition, different underlying models naturally lead to different results; in some cases, EAD can be explained by action potential reactivation, while in others, it is accompanied by intracellular Ca^2+^ release. Eventually, the accumulation of precise experimental data and trial and error in the analysis seem to be indispensable.

On the other hand, to elucidate the mechanism of cardiac function, Dr. Namekata et al. and Dr. Okada et al. employed a strategy of comparing different animal species [4,5]. To our knowledge, the normal beating heart is driven by a coupled-clock system in which Ca^2+^ release preceding the action potential is reflected in depolarization via a Na^+^–Ca^2+^ exchanger (NCX). Surprisingly, Dr. Namekata et al. pharmacologically excluded the need for an NCX in guinea pig heartbeat. Indeed, similar reports have previously been reported in cellular experiments [6]. Furthermore, the authors of the coupled-clock system themselves have recently reported in vivo observations demonstrating that the action potentials of pacemaker cells are heterogeneous, with Ca^2+^ release preceding the action potential, and the action potential preceding Ca^2+^ release [7]. It seems that the coupled clock is simply a phenomenological observation. Similarly, Dr. Okada et al. dismissed the high-scoring report stating that Tbx18 is the most critical transcription factor that characterizes pacemaker cells [8]. They reported that SHOX2 is the essential transcription factor for pacemaker cells when animal species, including humans, are considered [5].

The study of heart rhythm in this way has been a passion, and there are a wide variety of experimental and analytical methods. The most straightforward approach is to inhibit specific ion channels in the study of arrhythmias. For example, inhibition of human ether-a-go-go-related gene (hERG) channels is known to produce ventricular arrhythmias, and Dr. Tamura et al. reported that synthetic estrogen has a protective effect against hERG channel inhibition [9]. Dr. Shimizu et al. reported that the intravenous anesthetic propofol suppresses hyperpolarization-activated, cyclic nucleotide-gated (HCN) channels and is associated with bradyarrhythmia [10]. Furthermore, Dr. Flowler and Dr. Zissimopoulos reported a review of ventricular arrhythmias associated with ryanodine receptor 2 (RyR2) mutations [11]. RyR2 is a giant ion channel with ~560 kDa subunits forming a tetramer, and more than 350 mutations have been reported. Mutations, thus, need to be classified as indicated in this review. 

By the way, even though the molecular mechanisms of cardiac excitation and contraction are extremely well regulated, there is one heart region that is innately susceptible to arrhythmias: the pulmonary veins. The pulmonary veins are the source of the most frequent arrhythmia: atrial fibrillation. Bredeloux et al. have provided a review in this Special Issue on the history and latest findings in pulmonary vein research [12], and Okamoto et al. contributed new insights into the molecular mechanisms of pulmonary vein arrhythmias [13].

Finally, Dr. Omatsu-Kanbe discovered atypical cardiomyocytes (AMCs), which she reviewed in this Special Issue [14]. AMC is a new cell type, discovered in the cardiac myoblast removal fraction that was previously discarded in the experimental process. Because of their cell fusion ability, AMCs have the potential to play an active role in the healing process after myocardial injury, but their complete characteristics remain a mystery. It is deeply satisfying to see that the review manuscript on AMCs has finally been accepted for publication. New discoveries always have to fight against traditional standards. Still, researchers cannot stop fighting. We are proud to have produced a Special Issue that encourages new discoveries, such as that of AMCs. 
